# Spatial regularity of InAs-GaAs quantum dots: quantifying the dependence of lateral ordering on growth rate

**DOI:** 10.1038/srep42606

**Published:** 2017-02-13

**Authors:** T. Konishi, E. Clarke, C. W. Burrows, J. J. Bomphrey, R. Murray, G. R. Bell

**Affiliations:** 1 Centre for Collaborative Research, National Institute of Technology, Anan College, Anan, Tokushima, Japan; 2 EPSRC National Centre for III-V Technologies, Department of Electronic and Electrical Engineering, University of Sheffield, Mappin Street, Sheffield, S1 3JD, United Kingdom; 3 Department of Physics, University of Warwick, Coventry, CV4 7AL, United Kingdom; 4 Department of Chemistry, University of Warwick, Coventry, CV4 7AL, United Kingdom; 5 Department of Physics, Imperial College London, South Kensington Campus, London, SW7 2AZ, United Kingdom

## Abstract

The lateral ordering of arrays of self-assembled InAs-GaAs quantum dots (QDs) has been quantified as a function of growth rate, using the Hopkins-Skellam index (HSI). Coherent QD arrays have a spatial distribution which is neither random nor ordered, but intermediate. The lateral ordering improves as the growth rate is increased and can be explained by more spatially regular nucleation as the QD density increases. By contrast, large and irregular 3D islands are distributed randomly on the surface. This is consistent with a random selection of the mature QDs relaxing by dislocation nucleation at a later stage in the growth, independently of each QD’s surroundings. In addition we explore the statistical variability of the HSI as a function of the number *N* of spatial points analysed, and we recommend *N* > 10^3^ to reliably distinguish random from ordered arrays.

Molecular beam epitaxy (MBE) can be used to grow arrays of In(Ga)As QDs on GaAs(001) with very high crystalline quality, suitable for application in lasers, optical amplifiers and high-efficiency solar cells. By reducing the growth rate, larger QDs with longer wavelength emission can be grown. The number density of the QDs also decreases at low growth rate. Sparse arrays of QDs are valuable if single QDs are to be optically addressed, for example in quantum information applications. QD densities below 10 *μ*m^−2^ are desirable in order to resolve single QD emission within device structures^1^. Conversely, for use in QD laser structures the number density should be as high as possible in order to optimise gain. Here the wavelength could unintentionally shift due to accompanying decrease in QD size but is compensated by tuning capping (over-growth) conditions^2^. However, the number density of QDs (mean number per unit area) is merely a measure of central tendency and the *spatial distribution* of QDs could vary significantly at a given mean number density. The spatial pattern could be ordered (lattice-like), random, clustered (groups of closely-spaced QDs separated by large distances), or something in between these limits. These spatial patterns can be quantified by the Hopkins-Skellam index (HSI)^3^,^4^,^5^,^6^,^7^,^8^. It is known that when multiple layers of QDs are grown with thin spacer layers separating them, the lateral ordering of the QDs increases markedly from the seed layer through subsequent layers^9^. However, we are not aware of any detailed quantification of this effect nor of studies of the effects of MBE growth conditions on the spatial regularity of single-layer QDs.

Reflection high energy electron diffraction (RHEED) has been used to study MBE growth almost since its inception and gives valuable information about growth mechanisms. However, such information is necessarily spatially averaged. For example monitoring the surface step density, inferred from RHEED intensity changes, allows the transition between step-flow and 2D island growth modes to be studied. But quantitative information about island size distributions (ISDs) and growth morphology demands a real-space method. In this regard, scanning tunnelling microscopy (STM), both *in vacuo* (via quenching) and truly *in situ* (during MBE growth) has been predominant in the study of 2D island growth mechanisms where atomic-scale details are important. The study of larger 3D structures is readily achieved using *ex situ* atomic force microscopy (AFM). Insights into the mechanisms underpinning MBE growth can be gained by studying the morphology and statistics of islands below monolayer (ML) coverage^10^,^11^,^12^,^13^. For example, the concept of scaling behaviour of ISDs^14^ has been applied to both 2D islands^12^ and QDs^15^,^16^ in the InAs-GaAs(001) system.

Assuming that islands grow by absorbing atoms or molecules (“monomers”) migrating on the surface, an island’s size can be related to its local spatial environment on the surface through the idea of a capture zone (CZ)^17^. This is the region of the surface from which the island obtains new monomers. Defining each CZ as the region of the surface closer to the island in question than to any other island means that the CZ pattern of an array of small islands is simply the Voronoi tessellation of the points. The growth rate of an island should increase as the size of its CZ increases^18^. This connection has been studied theoretically^10^,^14^,^17^,^18^,^19^ for many years. The CZ distribution (CZD) is related to the ISD and may also show scaling behaviour^20^ but in the case of InAs-GaAs QDs, island coarsening at high growth temperatures breaks the connection between ISD and CZD^16^.

Under usual MBE growth conditions, the great majority of QDs are coherently strained, i.e. dislocation-free. However, dislocation nucleation can occur within QDs especially during long InAs growth times. The associated plastic relaxation results in a rapid increase of island size since adding InAs to a more strain-relaxed island costs less strain energy^21^ and the dislocated islands become more efficient at capturing monomers. In the present work we term such clusters “large 3D islands” (L3DI)^22^ and they may also break the ISD-CZD connection.

We recently performed an analysis of the spatial regularity of InAs-GaAs QDs^8^,^23^,^24^ imaged *during* MBE growth by fully integrated scanning tunnelling microscopy (STMBE)^25^. In that study we examined QDs grown at low temperature (430 °C) and compared their spatial distribution to that of nanometre-scale reconstruction domains in the wetting layer (WL) prior to QD nucleation. Here we examine QDs grown at more conventional temperature (500 °C) by *ex situ* AFM, and examine their spatial patterns as a function of growth rate, which can provide control over the QD density over 2 orders of magnitude^26^,^27^. We separately measure the regularity of QD and L3DI arrays to test the hypothesis that dislocation nucleation occurs randomly and independently of QD spatial environment. In addition, we analyse artificial random arrays of points in order to understand the reliability of the HSI as a function of the number of spatial points available in an array.

## Results and Discussion

### Growth rate dependence of 3D island arrays

**Figure 1:**
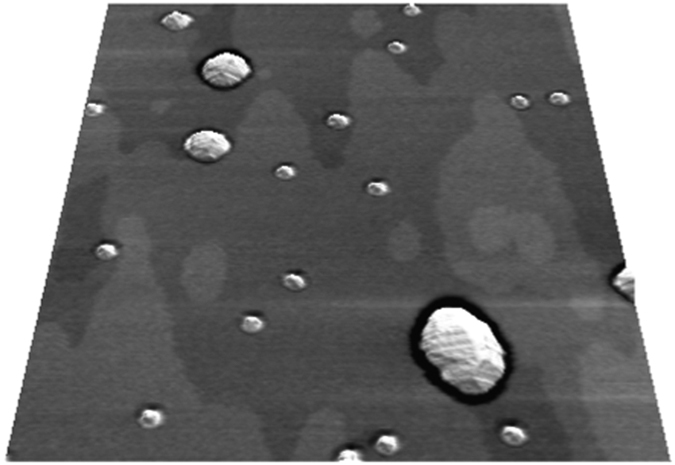
A 1 *μ*m × 1 *μ*m typical AFM image from an intermediate growth rate sample (3.58 × 10^−3^ ML s^−1^) rendered in 3D. The step-terrace structure of the WL is clear along with L3DIs and QDs.

We routinely observed atomically flat terraces separated by monolayer steps in the substrate. A typical 3D rendered image with suitably adjusted height scale is shown in Fig. 1, highlighting these terraces. The QDs and L3DIs appear white, and dark trenches at the base edges of both L3DIs and QDs are often discernible. Such structures have been previously observed in the InAs-GaAs(001) system^28^ at a comparable InAs growth rate of 3.58 × 10^−3^ ML s^−1^. Similar features in the analogous Ge-Si(001) system were explained energetically, with the trenches helping to relax strain at 3D island bases^29^, and they also appear in generic continuum simulations of strained epitaxy^30^.

**Figure 2:**
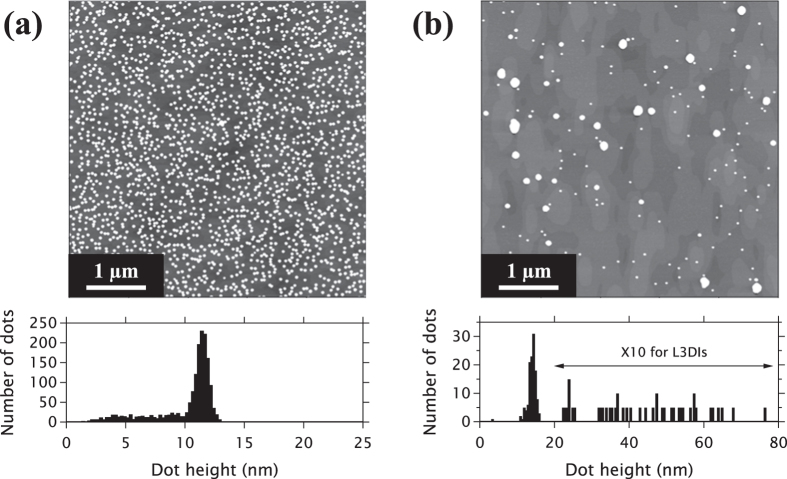
AFM images of QD arrays (upper panels) for (**a**) high growth rate (13.9 × 10^−3^ ML s^−1^) and (**b**) lower growth rate (1.53 × 10^−3^ ML s^−1^) samples. The images are 5 *μ*m × 5 *μ*m in size and the height scale is the same between the images to aid comparison. The lower panels show histograms of island centroid heights obtained from the respective AFM images.

**Figure 3:**
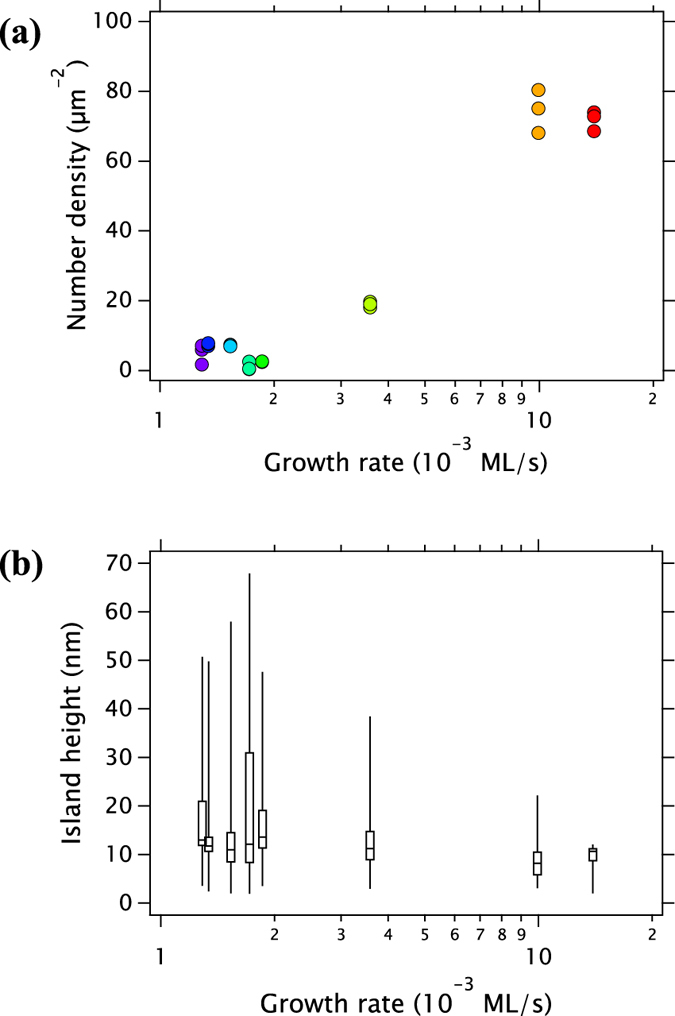
(**a**) Number density of all islands (QDs and L3DIs) as a function of growth rate. Circles for a given growth rate represent multiple images analysed from a single sample. (**b**) Box plot of island centroid heights with each horizontal line inside a box representing the median, box ends indicating the first and the third quartiles and whisker ends indicating 2% and 98% percentiles.

Further example AFM images acquired are shown in the upper panels of Fig. 2 for growth rates 13.9 × 10^−3^ ML s^−1^ and 1.53 × 10^−3^ ML s^−1^. The images clearly show the dependence of the dot density on the growth rate, with over 1500 dots present in the 5 *μ*m × 5 *μ*m scan area for the higher growth rate sample. In contrast, the lower growth rate has a density one-tenth this value with large irregularly shaped L3DIs very apparent. Figure 3(a) shows the 3D island number density (QDs + L3DIs) as a function of growth rate. As expected the density increases at higher growth rates^31^.

In order to automatically distinguish between QDs and L3DIs for thousands of islands, we explored several methods, but a simple height discrimination proved to be reliable for all samples. In the lower panels of Fig. 2 we show number-height distributions obtained from the respective AFM images (upper panels). For the higher growth rate sample, a peaked height distribution is revealed with a clear upper threshold of 15 nm.

For the lower growth rate sample, we find that a similar peaked distribution also occurs up to a threshold of 15 nm but islands with heights in excess of 20 nm are also present. The distribution of these larger islands is very broad. Inspection of the images reveals a reasonably clear distinction between islands above and below 15 nm in height, with the former being more irregular and significantly wider. This behaviour was also seen for other growth rates and so we used a simple height threshold to discriminate between L3DIs and QDs. Our HSI results are barely affected by small changes of threshold around the optimum value.

The statistics of height for all islands are presented using the box plot in Fig. 3(b). The upper whiskers extending to the higher side, prominent at lower growth rate, represent flat distribution of L3DIs. The quartile boxes correspond to the distribution peaks of normal QD seen in the histograms of Fig. 2. The median height of normal QDs maintains a value ~10 nm regardless of growth rate. The increase of number density at higher growth rates without a corresponding decrease of height can be explained by the increased incorporation of Ga into the QDs at higher growth rates^22^, i.e. the total volume in the QD array is larger^13^. The L3DIs are also larger for ultra-low growth rates, incorporating more deposited material.

### Spatial regularity of QDs and L3DIs

The lateral ordering of the QDs cannot be easily evaluated “by eye” in AFM images, but can be evaluated by computing the HSI (*I*_HS_, see Methods). Analysis was performed by using Fiji^32^ and MATLAB to segment the flattened AFM images, identify individual QDs and L3DIs and compute *I*_HS_ for all islands (QDs and L3DIs) and for L3DIs only. Typically, we could analyse images containing a few thousand QDs to give good statistical fidelity. Some smaller images are included in the analysis containing fewer QDs, even for the higher density arrays.

**Figure 4:**
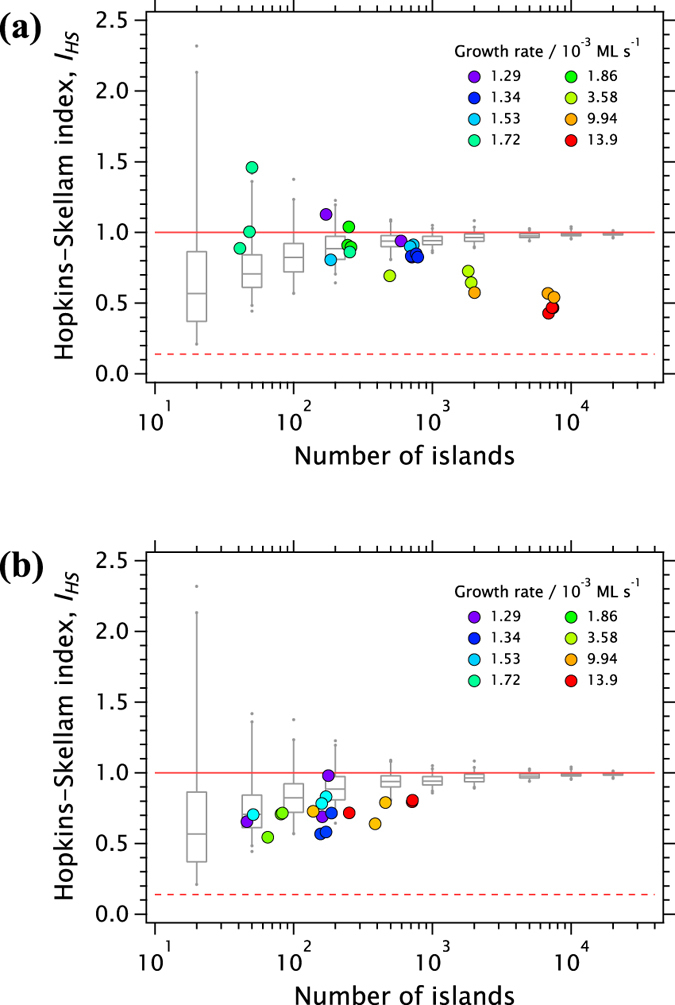
The HSI for (**a**) all islands (QDs and L3DIs) or (**b**) L3DIs only as a function of population. Circles for a given growth rate represent multiple images with different size but analysed from a single sample. The growth rate is represented by marker colours. Grey box plots indicate the HSI spread of artificial random island arrays and the dashed line indicates the theoretical minimum (*I*_HS_ = 5/36) for a perfect hexagonal close-packed array.

Figure 4(a) shows the HSI computed for all islands (normal QDs and L3DIs) plotted as a function of population (horizontal axis) and colour-coded by growth rate. Some individual data points represent smaller numbers of islands compared to others from the same sample (same growth rate): this is simply due to differences in AFM image sizes. The grey box plots represent the HSI range calculated for artificial pseudo-random arrays of islands: the boxes show mean and quartiles, the lines show the 2nd and 98th percentiles, and the dots show outliers (see Methods). These box plots allow the experimental data points to be compared to the *I*_HS_ distribution for arrays of different sizes with no lateral ordering.

At the higher growth rates, around 7000 islands are typically available in a single AFM image to calculate *I*_HS_, which clusters around 0.5 ± 0.1 (red and yellow points). Artificial random arrays with this number of points have *I*_HS_ narrowly distributed very close to unity. Clearly, the QDs with the highest growth rates of 13.9 × 10^−3^ ML s^−1^ and 9.94 × 10^−3^ ML s^−1^ are not randomly sited. The HSI values measured here are greater than theoretical minimum of 5/36 ≈ 0.14 corresponding to a perfectly ordered hexagonal array^8^, or 1/6 ≈ 0.17 for a perfect square array, but well below the range of values allowed for a random array. Hence we can say that the QD arrays at higher growth rates are partially laterally ordered. We surmise that this is due to the smaller spacing between QDs: interaction between QDs via their CZs is able to drive regularity in their spatial distribution even though QD nucleation is known to be a very rapid process locally^25^. Nucleation of a QD suppresses further nucleation nearby due to the local fall in density of available In adatoms and hence some spatial order arises.

Conversely, at ultra-low growth rates (<1.7 × 10^−3^ ML s^−1^, blue points) the maximum number of QDs in a AFM image to compute *I*_HS_ is around 700 and the measured *I*_HS_ spreads from ~0.8 to ~0.9. However, this is included in the range covered by random arrays for this number of islands. Two smaller images with around 200 islands are also included in the HSI range of random arrays. Hence it is not possible to distinguish these ultra-low growth rate QD arrays from randomly distributed points. This regime corresponds to widely-spaced QDs with no lateral ordering, consistent with a lack of interaction via CZs at the nucleation stage. It is interesting that no lateral ordering appears to be inherited from the substrate, e.g. through preferential nucleation of QDs near regularly-spaced terrace edges.

We also show intermediate growth rates (green points) for which a wider range of AFM image sizes has been employed. For the largest images, with around 2000 QDs, the measured HSI of ~0.7 is below the range of random arrays, again indicating partial lateral ordering of the QDs. However, smaller images with around 200 islands appear to show HSI values completely contained within the random array distribution. This confirms that the total number of islands analysed is important when determining the HSI and comparing it to a truly random spatial distribution. The overall trend for the InAs-GaAs QD arrays is that higher growth rates and correspondingly higher number density correspond to greater spatial ordering.

The HSI values counting L3DIs only are shown as a function of island numbers in Fig. 4(b), again colour coding by growth rate. The overall numbers are smaller due to the lower density of L3DI. At the highest growth rates there is a marginal indication that the experimental HSI values drop below the range for random islands. For all other growth rates, the L3DI HSI clearly lies within the spread of random values. Unlike normal QD, there is no trend of improved spatial ordering (reduced HSI) with increasing growth rate. Overall the L3DI appear to be randomly distributed independent of their “parent” QD array. A lack of lateral ordering is consistent with the idea that L3DIs arise by nucleation of a dislocation within a QD, a process which (unlike CZ fragmentation) does not depend on the neighbourhood of the QD. It is likely that strong local strain fields around QDs drive dislocation nucleation stochastically, with the long-ranged strain interaction between QDs being much weaker. Some L3DIs may also arise from the merging of two closely-nucleated QDs but it appears that these close pairs are not themselves spatially correlated in any way and so the resulting L3DI spatial distribution is random.

## Conclusion

Using the HSI we have quantified the lateral ordering of arrays of MBE-grown InAs-GaAs QDs as a function of growth rate at fixed coverage, substrate temperature and arsenic flux. At ultra-low growth rates, where the number density is very low, the QD arrays are spatially random even though the height distribution of QDs remains narrow. As growth rates increase the number density of QDs becomes higher and the QD arrays become significantly more ordered. This lateral ordering appears naturally as a consequence of QD-QD interaction during the rapid nucleation stage but does not approach HSI values consistent with truly ordered square or triangular arrays. In contrast, L3DIs show no lateral ordering, consistent with their formation via random dislocation nucleation or QD merging processes which occur independently of the L3DI neighbourhood. It would be interesting to measure HSI for QD arrays grown with techniques, such as Sb termination of the WL^33^, designed to maximise density. Another natural extension of this work would be to examine QD superlattices^9^ to quantify the lateral ordering as a function of layer number and spacing. More generally we have evaluated, using an efficient algorithm^8^, the HSI for artificial pseudo-random arrays with different total particle number, as a guide to the statistical robustness of the index for arrays of limited size. When the total number of particles analysed is below 10^3^, it becomes more difficult to distinguish random from partially ordered arrays.

## Methods

### Hopkins-Skellam index and artificial random arrays

The Hopkins-Skellam index (HSI) is a precise measure of spatial regularity which has been used mainly in the life sciences^3^,^4^,^5^,^6^,^7^. It allows ordered, random and clustered spatial distributions to be distinguished quantitatively. For example, the spatial distribution of beech tree canopy centroids has been found to be more regular than the corresponding distribution of tree trunks^4^. This can be explained by the tree crowns growing to maximise their access to light (phototropism and crown plasticity), efficiently filling the space between trunks and thereby increasing regularity. This is analogous to the CZ ideas outlined in this paper; competitive interaction among entities tends to increase their spatial regularity.

**Figure 5:**
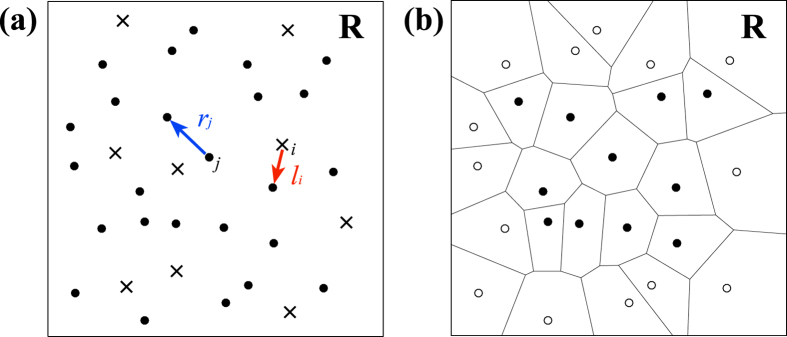
(**a**) Example spatial pattern of 25 islands (•_*j*_) illustrating *l*_*i*_ the distance from the *i*-th randomly selected location (x_*i*_) to the nearest island and *r*_*j*_ that from *j*-th island to the nearest island. (**b**) Voronoi tessellation of the example spatial pattern. Voronoi cells with open markers are excluded from the study region **R** because they touch the sides of **R**.

Let us consider the spatial arrangement of *N* islands in a region **R** (Fig. 5(a)). We define the distance from the *j*th island to its nearest neighbouring island as *r*_*j*_. In order to calculate the HSI, *I*_HS_, we plot *N* random locations in **R** and for the *i*th random location measure the distance *l*_*i*_ to the nearest island. Then the HSI is defined as


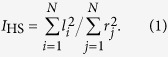


The HSI can be calculated by selecting exactly *N* random points in **R** and computing directly, but many repeated trials are required to ensure adequate randomness for a reliable HSI. However, accurate values can be calculated much more efficiently by using the Voronoi tessellation of the island array (Fig. 5(b)) together with the cumulative probability function for *l*_*i*_^8^. We employ this new method in the present work.

**Figure 6:**
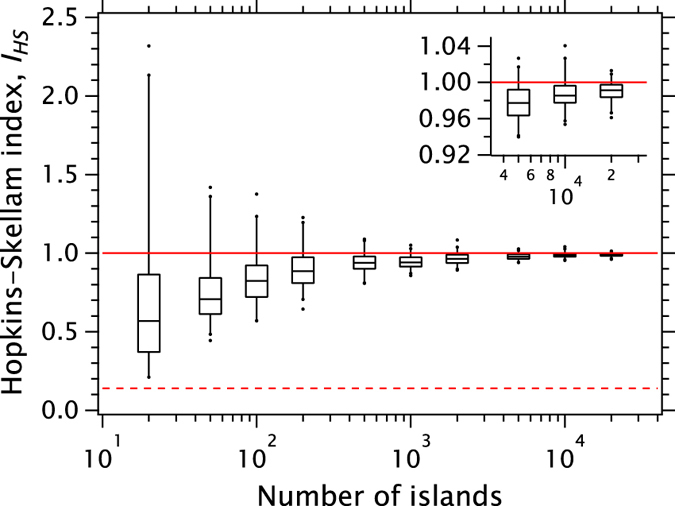
HSI of artificial random arrays of islands as a function of number of islands (100 arrays for each number). Boxes, whisker ends, dots represent quartiles, 2–98% percentiles, outliers, respectively. The ideal HSI is unity but due to statistical fluctuations the spread of points becomes larger, and biased towards lower HSI values, for smaller island numbers. Solid line (*I*_HS_ = 1) indicates perfectly random pattern and dashed line (*I*_HS_ = 5/36) indicates the theoretical minimum for the hexagonal close-packed array.

The statistical fidelity of the HSI depends on the number of islands (points) in **R** and to quantify this point, in Fig. 6, we show the computed HSI for artificial pseudo-random arrays as a function of *N*. For each *N*, 100 values of *I*_HS_ were calculated to show the distribution about the ideal value of 1. When *N* ≥ 10^3^ the computed values cluster closely around values slightly smaller than 1. As the number of islands falls, the distribution of *I*_HS_ values calculated for a given *N* becomes broader, as expected. However, the mean *I*_HS_ also drops well below unity while the right-skewness of the distribution increases markedly. This means that it is more difficult to unambiguously detect spatial ordering when the number of islands analysed is small, *N* ≈ 10^3^ or fewer. In other words, a few hundred points with I_*HS*_ < 1 can actually be part of a distribution which is genuinely random in a wider field of view.

### QD growth and AFM measurements

For the experiments, arrays of QDs were grown using conventional MBE and were left uncapped. The 2D-3D transition time *t*_*crit*_ was monitored using RHEED and the growth terminated after a total growth time 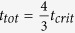, corresponding to an InAs coverage of approximately 2.25 ML. The substrate temperature (500 °C) and As_2_ flux were fixed for all samples. The growth rate (growth time) varied from 13.9 × 10^−3^ ML s^−1^ (160 s) to 1.29 × 10^−3^ ML s^−1^ (1760 s) and 8 separate samples with different growth rates were analysed. The native oxide of InAs-GaAs is quite stable and allows high resolution imaging of the surface even after long atmospheric exposure.

AFM measurements were made using two Asylum Research MFP-3D microscopes. Images were obtained in tapping mode using standard silicon probes and cantilevers, with a image size of 5 *μ*m × 5 *μ*m or 10 *μ*m × 10 *μ*m. In all cases the pixel density was kept sufficiently high to unambiguously identify the QDs even when a single image contained more than 10^3^ QDs. Image levelling was done using an iterative line-by-line first-order subtraction process with masking (the “magic mask” method in the Asylum Research software).

## Additional Information

**How to cite this article**: Konishi, T. *et al*. Spatial regularity of InAs-GaAs quantum dots: quantifying the dependence of lateral ordering on growth rate. *Sci. Rep.* **7**, 42606; doi: 10.1038/srep42606 (2017).

**Publisher's note:** Springer Nature remains neutral with regard to jurisdictional claims in published maps and institutional affiliations.
